# Robust and High-Performance Machine Vision System for Automatic Quality Inspection in Assembly Processes

**DOI:** 10.3390/s22082839

**Published:** 2022-04-07

**Authors:** Fabio Frustaci, Fanny Spagnolo, Stefania Perri, Giuseppe Cocorullo, Pasquale Corsonello

**Affiliations:** 1 Department of Informatics, Modeling, Electronics and Systems Engineering, University of Calabria, 87036 Rende, Italy; f.frustaci@dimes.unical.it (F.F.); f.spagnolo@dimes.unical.it (F.S.); giuseppe.cocorullo@unical.it (G.C.); p.corsonello@unical.it (P.C.); 2 Department of Mechanical, Energy and Management Engineering, University of Calabria, 87036 Rende, Italy

**Keywords:** geometrical model, machine vision, automatic in-line inspection, hardware–software co-design, field programmable gate array systems-on-chip, assembly process

## Abstract

This paper addresses the problem of automatic quality inspection in assembly processes by discussing the design of a computer vision system realized by means of a heterogeneous multiprocessor system-on-chip. Such an approach was applied to a real catalytic converter assembly process, to detect planar, translational, and rotational shifts of the flanges welded on the central body. The manufacturing line imposed tight time and room constraints. The image processing method and the features extraction algorithm, based on a specific geometrical model, are described and validated. The algorithm was developed to be highly modular, thus suitable to be implemented by adopting a hardware–software co-design strategy. The most timing consuming computational steps were identified and then implemented by dedicated hardware accelerators. The entire system was implemented on a Xilinx Zynq heterogeneous system-on-chip by using a hardware–software (HW–SW) co-design approach. The system is able to detect planar and rotational shifts of welded flanges, with respect to the ideal positions, with a maximum error lower than one millimeter and one sexagesimal degree, respectively. Remarkably, the proposed HW–SW approach achieves a 23× speed-up compared to the pure software solution running on the Zynq embedded processing system. Therefore, it allows an in-line automatic quality inspection to be performed without affecting the production time of the existing manufacturing process.

## 1. Introduction

The production line of manufacturing processes is typically divided into consecutive separated steps that are demanded to robots and/or automated machines, which can accomplish their tasks quicker than a human operator can, with a higher accuracy and in a safer mode [1]. However, the whole automation-based flow does not have a complete feedback control system; thus, the production line imposes some checkpoints aimed at verifying the correct operation of the single production step. Such verification procedures typically consist of a visual inspection of the item within the production line and in checking its geometrical compliance with the particular requirements. Very often, the check is performed by a human operator who manually operates on the item with the help of specialized instruments, such as microcenters and calibers. A typical example is the geometrical compliance check of an assembled item where its components are welded together by a welding robot [2]. The human-based compliance check has some obvious drawbacks: it is time consuming and it requires the line to stall for the time required by the checking operation. Moreover, as a consequence, it can be applied on one single or a few samples over a set within the production lot, preventing the collection of useful data about the manufacturing process that may be used, as an example, for a predictive maintenance based on big data analytics.

Computer vision systems (CVSs) have emerged as effective alternatives to human-based inspections [3,4,5]. Indeed, they represent an effective methodology to build a digital twin of the item to be analyzed, i.e., a digital avatar or a cyber–physical equivalent that can be used in real time in parallel with its physical counterpart [6]. The digital twin of the item is updated in real time by the data that are continuously gathered by the sensors (i.e., the cameras) and it allows a continuous analysis of the virtual item in a more accurate and faster way than usually required by the physical item [7].

CVSs enable an in-line inspection check that can be performed without interfering with the regular flow of the production line and, hence, without requiring a periodical production stall. Moreover, a CVS is not affected by possible errors that a human operator may commit and its high execution speed allows inspecting much more assembled items. Depending on the assembled item to be inspected, a CVS has to face particular challenges. As an example, the image segmentation process aimed at extracting the features of interest can be difficult due to poor luminosity, color homogeneity of the object to analyze, noisy background, and/or surface irregularity. Another typical challenge arises when the geometrical check needs a three-dimensional optical digitizing. Finally, in order to avoid interferences with the existing manufacturing line and its production speed, the CVS has to assure a high computational speed. Existing works deal with some of these problems by proposing strategies that unfortunately cannot be generally applied. As an example, the approaches proposed in [8,9] exploit forward and backward lighting to eliminate shadows and to enhance edges, respectively, and to artificially change the color of the object of interest. This is highly required by any machine vision system because the illumination noise and the reliability of the measurement are correlated: the higher the noise (i.e., the more the illumination deviates from the assumed one) the higher the error in the measurement. The three-dimensional optical digitization of the item proposed in [10,11] uses rotating tables. This kind of solution may not be applicable when the room available to integrate the CVS with the manufacturing line is constrained and/or inaccessible. The CVSs described in [12,13,14] are suitable to detect geometrical deviation of an object from a reference pattern, but they are able to detect only the movements of the item along a plane parallel to the video camera. As deeply discussed in [15,16,17], machine learning and deep learning techniques are also widely adopted in CVSs for inspection tasks. However, such techniques aim to classify surface imperfections of the inspected item and they are not used to detect position shifts from a reference point. Furthermore, they require a large training image data set with known information of all the possible defects that may not be available and/or easily collected. Finally, in order to deal with noise due to irregular illumination, some papers [15,18,19,20] propose to iterate the computation of the CVS over a large set of consecutive frames. Indeed, random illumination noise can affect the correctness of the measure. By iterating the procedure on several consecutive frames and by averaging the resulting measurements, the effect of noise is averaged too and, thus, the final measured value is more reliable. However, this strategy increases the computational time of the CVS and makes the use of a compact and high performance specialized hardware device highly desirable to meet the production timing and physical space constraints [21].

To solve the aforementioned limits, in [22] we proposed a geometrical model of the 3D space that can be embedded in a CVS for a flexible, precise, and low-cost in-line geometrical inspection of the assembly processes. As a case study, it was applied to the geometrical compliance check of a real catalytic converter assembly process. The geometrical modeling makes the CVS able to detect planar, translational, and rotational shifts of the flanges welded on the central body of the catalytic converter by using two-dimensional elaborations of an input image. To improve measurement accuracy, several consecutive captured frames are processed and averaged by the proposed algorithm, thus reducing noise effects. As a drawback, the total computational time of course increases and may eventually exceed the available time slot. This problem is even more significant if it is considered that such procedures usually run on a single board computer (SBC) with limited hardware resources [22]. This paper extends the previous work by proposing the design of an embedded system based on a heterogeneous multiprocessor system-on-chip (MPSoC) hosting a general-purpose multicore processor and a reconfigurable programmable logic platform based on a field programmable gate array (FPGA). To this purpose, the geometrical inspection algorithm was analyzed and a detailed timing profiling of its computational steps was developed. Then, the most timing consuming tasks were accelerated by an on purpose designed hardware architecture realized in the programmable logic portion of the chip; whereas the remaining steps were executed by software routines by the embedded processor.

The proposed embedded system was implemented using the PYNQ-Z2 development board, equipped with the Xilinx XC7Z020-1CLG400C SoC. The automatic inspection of the geometrical compliance of the flanges of catalytic converters is referred to as the application of interest. Our results show that, when supported by the system described here, the whole computation is performed within a total processing time of only 1.3 s, thus being 23 times faster than the pure software solution. Due to this, the proposed methodology can be successfully exploited to design CVSs suitable for integration into a manufacturing line without interfering with the production process.

The remainder of the paper is organized as follows: Section 2 outlines the specifications of the assembly process related to the catalytic converter; Section 3 discusses the image processing and the adopted geometrical model; Section 4 describes the proposed architecture of the heterogeneous embedded system with a particular emphasis on the FPGA-based hardware accelerator; finally, some conclusions are drawn in Section 5. 

## 2. The Catalytic Converter Assembly Process

**Figure 1:**
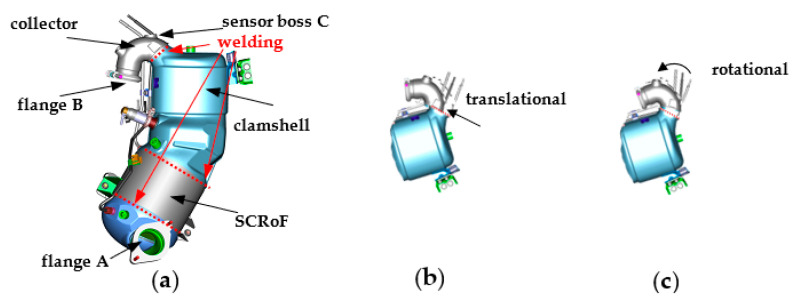
The catalytic converter: (**a**) the model; (**b**) possible translational; and (**c**) the rotational shift of the collector (courtesy of Marelli Europe SPA Green Technology Systems, Caivano, Italy).

**Figure 2:**
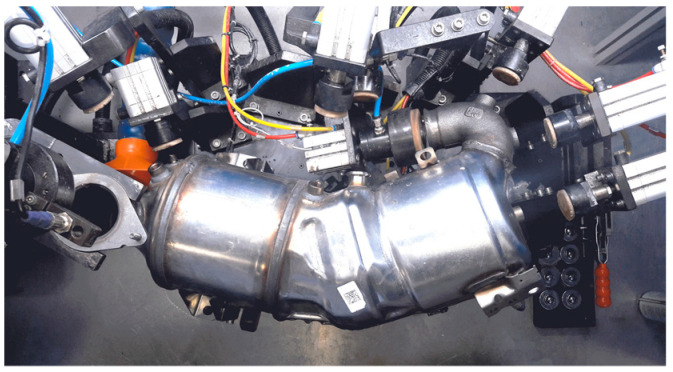
The assembled catalytic converter into the quality cell (courtesy of Marelli Europe SPA Green Technology Systems, Caivano, Italy).

Figure 1a illustrates a catalytic converter of an actual manufacturing process. It is composed of two collectors, two clamshells, and a selective catalytic reduction on filter (SCRoF) chamber. The two collectors are welded to their respective clamshell by an automated metal active gas (MAG) welding process, which is demanded to an automated robot. Once assembled, the item is placed in a quality cell, as shown in Figure 2, for a leakage test that takes about one minute. If process imprecisions occur, the collector B may experience a translational and/or a rotational shift from its reference position, as schematized in Figure 1b,c. The aim of the inspection task is to check the geometrical position of the flange B. In order to avoid interferences with the production line, the inspection can be demanded to a CVS running when the catalytic converter is fixed in the quality cell, just before the beginning of the leakage test.

As visible in Figure 2, the room available to integrate the system (including the video camera, the illumination source, the computing platform, etc.) with the existing quality cell is very constrained. This forces the system to be flexible and able to operate with reduced degrees of freedom (e.g., the camera and the illumination source cannot be arbitrarily positioned, the computing platform should have a reduced size, etc.). Due to the limited room, it is not possible to place a video camera capturing frames of the flange B. Nevertheless, the focus of the CVS can be moved to the sensor boss C [22]. Indeed, the latter does not undergo any welding process but it is monolithically realized on the collector. As a result, any geometrical noncompliance of the flange B is then reflected on the sensor boss C.

## 3. The Image Processing and the Geometrical Model

The algorithm at the basis of the proposed system consists of two steps: the image segmentation, which extracts the region of interest (ROI), and the feature extraction that detects translational and rotational shifts. 

### 3.1. Image Segmentation

**Figure 3:**
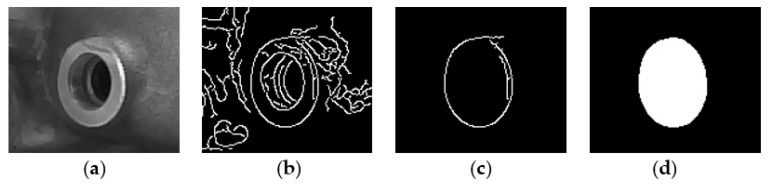
Outputs of the proposed image segmentation procedure on the sensor boss: (**a**) image conversion, (**b**) ROI filtering; (**c**) contour selection; (**d**) morphological filtering.

The image segmentation consists of the following main steps:

1. The color image acquisition from the video camera (GeTCameras Inc., Eindhoven, The Netherlands); 640 × 480 image resolution was used in our experiments;
2. The image conversion and cropping. It converts the input image from the RGB color space into the 8-bit grayscale domain. Moreover, it crops a 220 × 220 region of the original image, which contains the profile of the sensor boss C, to reduce the computational complexity;
3. The ROI filtering removes noise and detects the edges of the sensor boss. Firstly, a 5 × 5 median filter removes the noise from the image. Successively, a Canny filter with lower and higher thresholds of 10 and 50, respectively, detects the edges [23]. The filter dimension and its thresholds are set empirically. The Canny filter was chosen since it is well known as one of the most robust processing methods for edge detection [24,25,26,27];
4. The contour selection aims at selecting only the edge related to the external profile of the sensor boss. The inevitable irregularities on the surface of the collector, as well as noise in the illumination conditions over time, cause the detection of several edges that are not of interest. The goal is to find a reasonable procedure to select just the contour related to the profile of the sensor boss. Towards this aim, all of the contours in the filtered image produced by the previous step are stored into a data structure. The contour with the largest length is then selected as the one that most likely is related to the boundary of the sensor boss;
5. The morphological filtering of the selected contour. It is important that the selected contour delimits a closed area in order to robustly discern, in the image, the area of the sensor boss from the other non-relevant regions. Due to noise, such a condition may be not satisfied. Moreover, the selected contour may still contain some irregularities that can affect the precision of the sensor boss detection procedure. To remove such non-idealities, a 3 × 3 morphological dilatation filtering is used that thickens the selected contour so that any possible gap can be filled, and, most likely, the contour can delimit a closed area. Successively, the pixels that are inside the identified area are set to the same value (i.e., 255, corresponding to white in an 8-bit grayscale image). A 3 × 3 morphological erosion filtering restores the original size of the detected enclosed area. Afterwards, two consecutive erosion and dilation morphological filtering, with their larger 21 × 21 kernel sizes, eliminate any other possible fringes outside the sensor boss area from the segmented image. Figure 3 illustrates the intermediate outputs of the above discussed steps.

### 3.2. Features Extraction

The segmented image is then processed to extract features of interest. Firstly, a connected component analysis is performed. Then, the central coordinates ($X_{P},\;Y_{P}$) in the 2D camera plane are calculated as shown in Equation (1):

(1) $$m_{ji}=\sum_{x,y}\left[P\left(x,y\right)\cdot x^{j}\cdot j^{i}\right]\;\;X_{P}=\frac{m_{10}}{m_{00}},\;Y_{P}=\frac{m_{01}}{m_{00}},$$

where P(x,y) is the value of the generic pixel belonging to the segmented area (i.e., with the same label) at the (x,y) coordinates. The procedure described so far is made more robust by iterating it over a certain number of consecutive captured frames. The coordinates ($X_{P},\;Y_{P}$) are then calculated by averaging the obtained results [18]. The appropriate number of processed frames may change according to the illumination setup: more frames are required with a lower illumination condition. For the specific application, we experimentally verified that 50 consecutive frames are enough to obtain a fairly robust parameters extraction. 

Considering the pinhole camera model [28], the coordinates ($X_{P},\;Y_{P}$) can be mapped into the coordinates ($X_{w},\;Y_{w}$) in the real world, on a plane that is parallel to the camera plane, by means of Equation (2), where $Z_{0}$ is the camera–object distance in the real world and F is the focal length (in pixels) of the camera:

(2) $$\;\left(\begin{array}{c} X_{P}\\ Y_{P} \end{array}\right)=\frac{F}{Z_{0}}\cdot \left(\begin{array}{c} X_{W}\\ Y_{W} \end{array}\right).$$

Consequently, Equation (3) can be applied when an imprecise welding process causes the sensor boss translational shifts from a reference point A to a point B on a plane parallel to the camera plane,

(3) $$\left(\begin{array}{c} \Delta X_{W}\\ \Delta Y_{W} \end{array}\right)=\left(\begin{array}{c} \left|X_{W,B}-X_{W,A}\right|\\ \left|Y_{W,B}-Y_{W,A}\right| \end{array}\right)\;=\frac{Z_{0}}{F}\cdot \left(\begin{array}{c} \left|X_{P,B}-X_{P,A}\right|\\ \left|Y_{P,B}-Y_{P,A}\right| \end{array}\right)=\frac{Z_{0}}{F}\left(\begin{array}{c} \Delta X_{P}\\ \Delta Y_{P} \end{array}\right),$$

with ($\Delta X_{W},\;\Delta Y_{W}$) and ($\Delta X_{P},\;\Delta X_{P}$) being the translational shifts of the sensor boss center in the real world and in the camera plane, respectively, along the *x*- and *y*-axes. 

**Figure 4:**
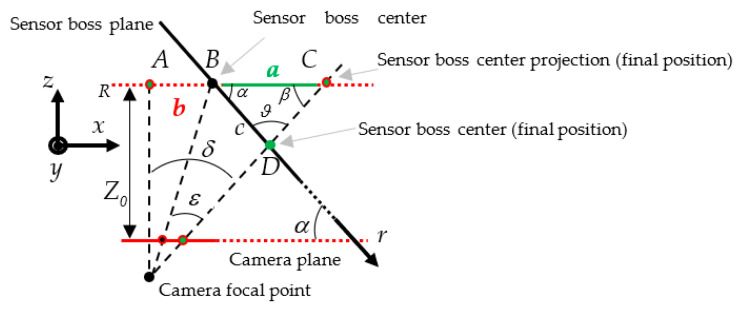
Geometrical model for translational shifts.

To improve the global accuracy, we adopted the more realistic geometrical model depicted in Figure 4. There, the center of the sensor boss experiences a translational shift on a plane not parallel to the camera plane. The values of the following parameters can be considered as inputs to the proposed system (they can be measured on the actual set-up and position of the video camera inside the quality cell): α is the angle between the camera and the sensor boss planes; $Z_{0}$ is the distance between the sensor boss center and the camera plane; b is the distance between the sensor boss center and the point A (i.e., the point with the same distance from the image plane as the expected ideal position B, but aligned with the camera focal point). An imprecise welding process may cause a shift of the sensor boss center from the expected ideal position B to a point D. Let us define with r and c the direction of such a shift and its magnitude, respectively. By detecting the sensor boss center coordinates in the image plane, Equation (2) can be applied to measure the value a. Since the sensor boss and the camera planes are not parallel, a is not the final shift magnitude because point C is only the projection of point D on the plane R. Under the realistic assumption that $Z_{0}$ is much larger than F, the measure of the actual shift c in the real world can be found by Equation (4):

(4) $$\begin{array}{l} \delta =arctg\frac{a+b}{Z_{0}}\\ \beta =\frac{\pi }{2}-\delta =\frac{\pi }{2}-arctg\frac{a+b}{Z_{0}}\\ \vartheta =\frac{\pi }{2}+\delta -\alpha =\frac{\pi }{2}+arctg\frac{a+b}{Z_{0}}-\alpha \\ c=a\cdot \frac{sin\beta }{sin\vartheta }=a\cdot \frac{sin\left(arctg\frac{a+b}{Z_{0}}\right)}{cos\left(arctg\frac{a+b}{Z_{0}}-\alpha \right)} \end{array}$$

**Figure 5:**
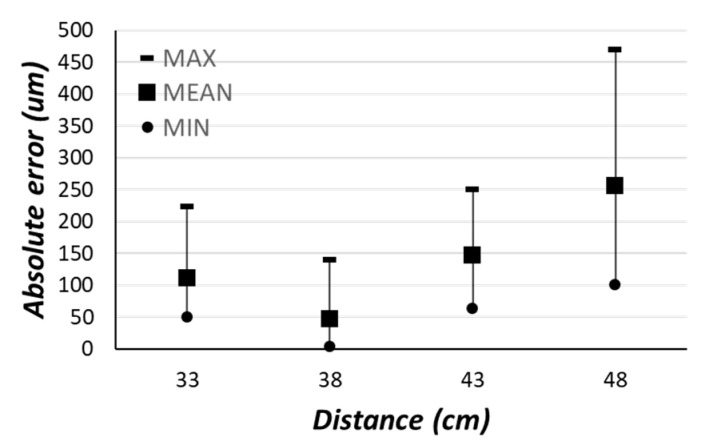
Experimental measurements for different distances between the camera and the sensor boss.

**Figure 6:**
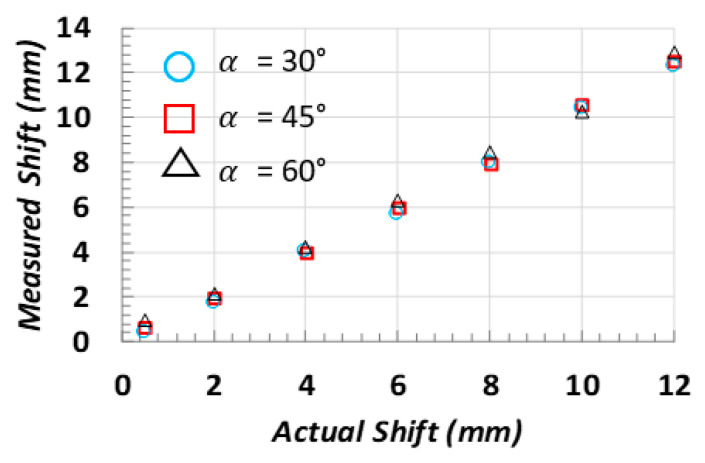
Obtained measurements applying the proposed model as a function of the translational shift extent and the angle between the camera and the sensor boss planes (b = 5 cm and $Z_{0}$ = 48 cm).

To experimentally validate the above model, the item under test and the video camera were placed on an optical bench. To emulate translational shifts, the camera was subjected to micro-movements through a mechanical carriage that allowed controllable and measurable movements with tenth-of-millimeter resolution. Figure 5 depicts the maximum, minimum, and mean absolute error measured through experimental tests on several manufactured items and for different distances between the camera and the sensor boss. It is worth noting that the obtained errors are of the order of microns. Figure 6 depicts the values of the measured vs. the exact shifts for different values of α. As visible, the proposed model is able to measure the shift of the sensor boss center with a high precision: the obtained maximum error was found to be below the millimeter.

**Figure 7:**
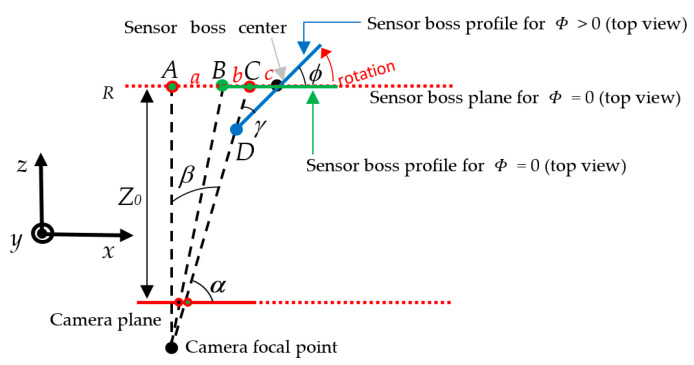
Geometrical model for rotation shifts.

**Figure 8:**
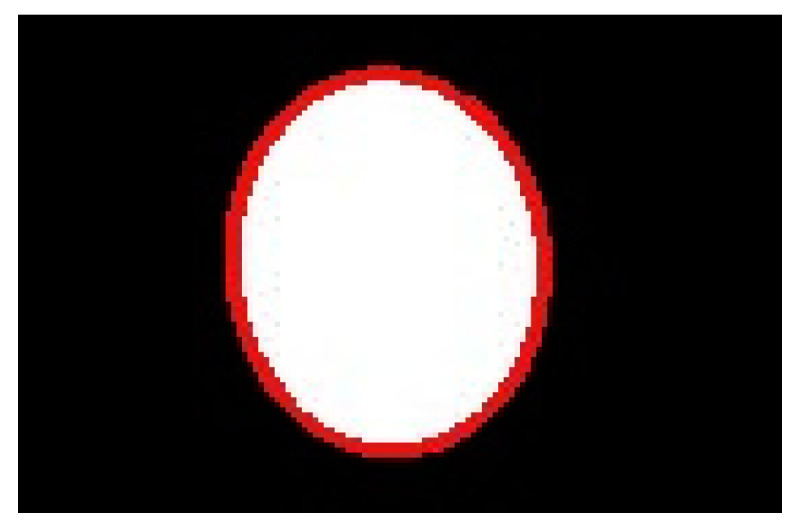
Elliptical matching (in red) of the sensor boss surface (in white).

Finally, the model was able to detect possible rotations of the sensor boss boundary. Such displacements can occur along an axis passing through the sensor boss center. The geometrical model was enhanced, as depicted in Figure 7, where ϕ is the rotation angle to detect, $Z_{0}$ is the distance between the camera and the sensor boss planes for ϕ = 0, and B (D) is the point representing the left border of the sensor boss boundary for ϕ = 0 (ϕ > 0). According to the camera pinhole model, points D and C are projected onto the image plane in the same position. Due to the rotation, the sensor boss on the image plane assumes an elliptic shape with an eccentricity depending on ϕ, so the length of the segment c can be obtained by finding one semi-axis of such an ellipse. Towards this aim, a synthetic ellipse is drawn in the image tracing the sensor boss boundary, as depicted in Figure 8, and the lengths of the semi-axes (in pixels) are obtained. The latter is then converted into a measure in the real world by Equation (2). Afterward, the rotation angle ϕ can be calculated by Equation (5), derived by the geometrical model of Figure 7:

(5) $$\begin{array}{l} \beta =arctg\frac{\left|b+a\right|}{Z_{0}}\alpha =\frac{\pi }{2}-\beta \frac{c}{sin\gamma }=\frac{b+c}{sin\left(\pi -\alpha \right)}\\ \gamma =arcsin\frac{c\cdot sin\left(\pi -\alpha \right)}{b+c}\;\\ \phi =\pi -\gamma -\left(\pi -\alpha \right)=\alpha -\gamma \; \end{array}$$

**Figure 9:**
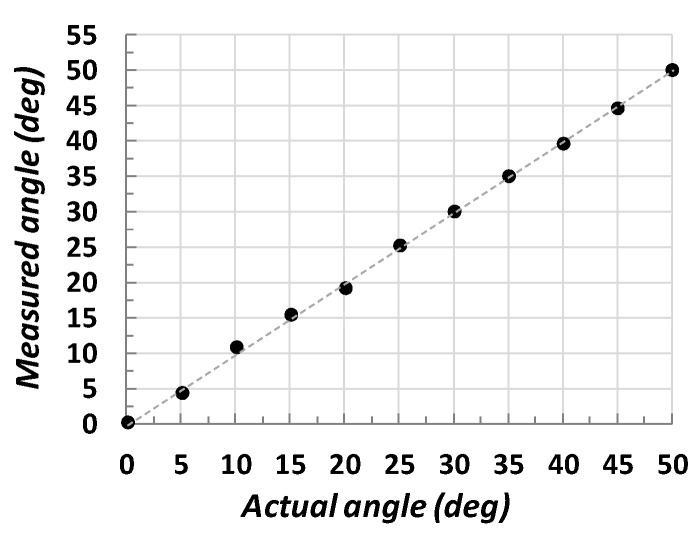
Obtained measurements applying the proposed model to detect rotations (Equation (5)).

Some of the obtained experimental results are depicted in Figure 9, which shows that the proposed methodology is able to detect the rotation angle ϕ with a maximum error below 1 sexagesimal degree.

## 4. The Hardware System

We implemented the designed CVS on a Xilinx Zynq XC7Z020-1CLG400C heterogeneous SoC (AMD-Xilinx Technology Ltd., Cambridge, UK) because it is a compact and flexible hardware platform that also allows multiple geometrical inspection processes running in parallel. Such a device provides a dual-core Cortex-A9 processor, also running the PetaLinux v2.4 operating system, and a programmable logic area of about 85 K logic cells.

As discussed in Section 2, the assembled catalytic converter is expected to remain inside the quality cell for no longer than one minute. The automatic geometrical inspection should be activated just after the operator places the item in the constrained position, shown in Figure 2. Once the leakage test starts, sensor bosses and flanges are plugged by hydraulic pistons; thus, they cannot be inspected anymore. Therefore, the CVS has to complete the automatic inspection in less than 10 s, to avoid an excessive slowdown of the production step.

**Figure 10:**
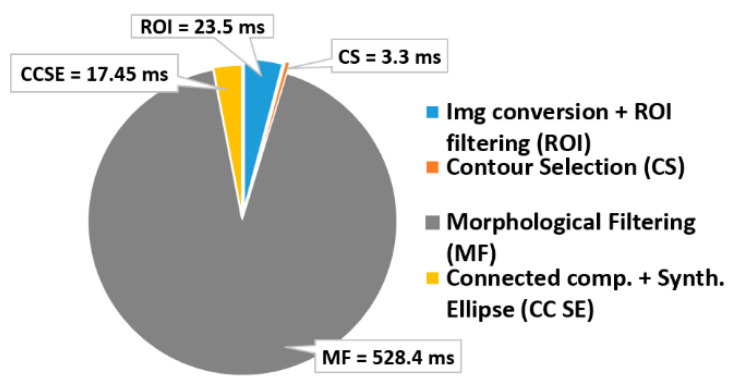
The time breakdown of the pure software execution of the geometrical inspection algorithm.

The pure software implementation of the algorithm described in Section 3 requires 0.573 s per frame, when it runs on the Zynq embedded ARM processing system. Therefore, processing at least 50 consecutive frames to obtain a reliable inspection of the item under test would lead to a timing specification violation. This pure software implementation evidently does not meet the above-mentioned inspection time constraint. For this reason, we adopted a hardware/software co-design approach: custom hardware accelerators execute the most time-consuming computational steps, whereas the less critical operations rely on the general-purpose processor of the Zynq SoC. Figure 10 shows the time breakdown of the pure software elaboration of a single frame. It can be noted that the morphological filtering is responsible for almost 92% of the computational time, whereas the image conversion and the ROI filtering operation account for the 4.1% of the total latency. Hence, these represent the time-critical computational steps that deserve to be hardware implemented.

**Figure 11:**
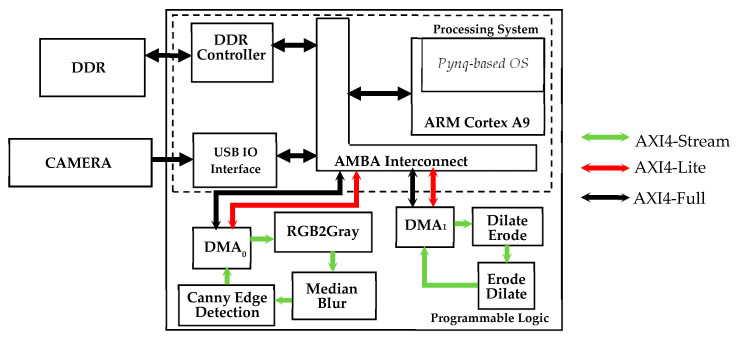
Top-level architecture of the designed embedded system.

Figure 11 depicts the architecture of the proposed embedded system. The SoC device is formed by the processing system (PS), hosting the Cortex-A9 processor, and the programmable logic (PL), where the FPGA is located. Subsystems implemented in the PL run with 100 MHZ clock frequency. The operating system running on the PS manages the USB communication with the video camera. The PS has direct access to the DDR memory controller, so the operation of storing the captured frames into the DDR memory is controlled by a software routine. Besides running the less critical computational steps of the algorithm, the PS also has to program and activate two direct memory access modules, DMA_0_ and DMA_1_, which regulate the input/output data flow between the hardware accelerators accommodated into the PL and the external DDR memory. For this task, the software running on the PS uses the port M_GP0 of the processor through the AXI4-Lite protocol [29].

**Figure 12:**
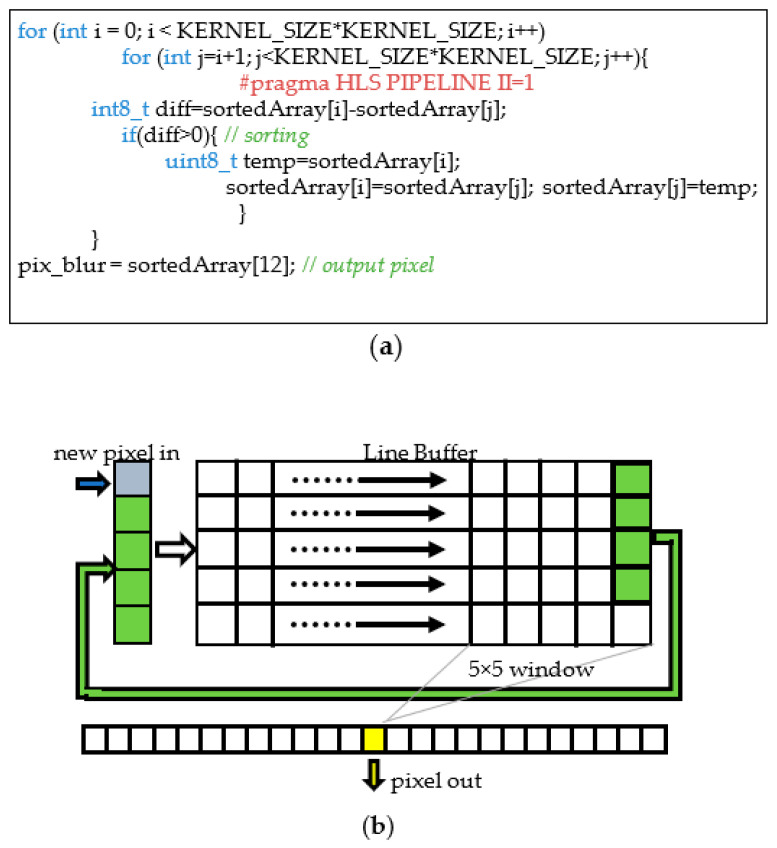
The design of the median blur hardware module: (**a**) excerpt of the C++ HLS code; (**b**) the resulting synthetized circuit architecture.

When not directly available as synthesizable intellectual properties (IPs), the required hardware accelerators have been described using the C++ high-level language and then synthesized through the Xilinx high-level synthesis (HLS) Vivado Tool [30]. Once the captured frame is stored in the DDR memory, the DMA_0_ is configured to transfer it to the first hardware accelerator through the AXI4-Stream protocol. As dictated by the DMA_0_ module interface, the input data flow consists of a stream of 32-bit words, each one containing a 24-bit RGB pixel. The stream enters the module RGB2Gray and flows through the modules median blur and canny edge detection. Eventually, it turns back into the DMA_0_ that provides the transfer of the result of the image conversion and ROI filtering operation into the DDR memory. As an example, Figure 12 shows an excerpt of the C++ HLS description of the developed median blur module and the synthesized hardware performing the required sorting of the pixels belonging to the 5 × 5 window. The incoming 8-bit grayscale input pixels are stored into a line buffer. The latter has a width of 220 (i.e., the number of pixels within the image rows) and a depth of 5 (i.e., the size of the chosen kernel). At each clock cycle, the pixels in the line buffer are right-shifted in a circular way and a new incoming pixel enters the buffer, as depicted in Figure 12b. At the same time, a 5 × 5 window of pixels is selected. The latter is rearranged into a linear vector composed of 5 × 5 8-bit registers that is used as data structure to perform the sorting algorithm described in Figure 12a and required by the median filtering operation. The *#pragma HLS PIPELINE II=1* assures that the synthetized hardware is provided with a pipeline sustaining a unitary throughput (i.e., a new result is outputted every clock cycle). The pipeline reduces the clock period, thus making the computation faster. Moreover, with a unitary throughput, the data stream coming from the previous RGB2Gray module does not require to be stalled, which is beneficial for the overall timing performance of the embedded system. Eventually, the pixel placed in the median position of the sorted array is selected as the filter output.

**Figure 13:**
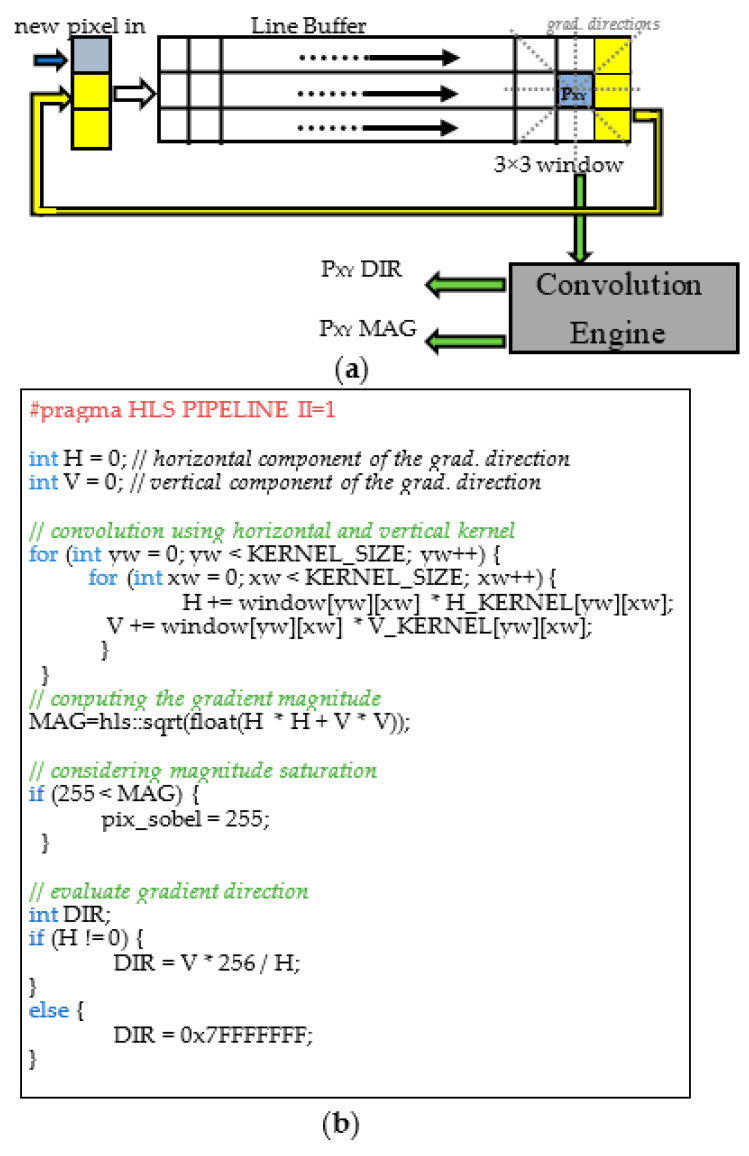
(**a**) The hardware architecture for the gradient calculation; (**b**) excerpt of the C++ HLS code.

**Figure 14:**
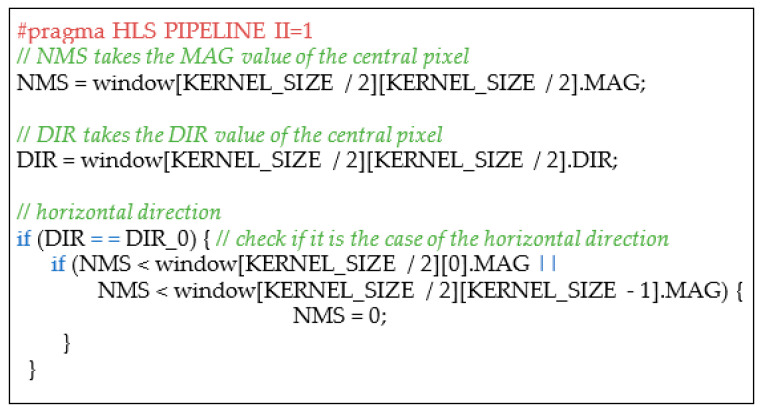
Excerpt of the C++ HLS description of the non-maximum suppression module.

The Canny module was designed following the same approach. The module is responsible for the following main consecutive computational steps: Sobel filtering, non-maximum suppression, double thresholding and Hysteresis comparison [23]. The Sobel filtering calculates the gradient magnitude and direction for each input pixel. Pixels that have a high gradient magnitude are considered to belong to an edge. The gradient is calculated based on the pixel intensity values: for each pixel, the direction along which there is the maximum intensity variation (direction) and the amount of such a variation (magnitude) are calculated. Practically, the gradient computation is equivalent to two convolutional operations between a 3 × 3 pixel window and two constant kernels of the same dimension. As for the median filter, the two convolutional operations can be accelerated by a hardware architecture performing the required computations on the 3 × 3 pixels in parallel. The output pixels of the median filter enter a line buffer described in Figure 13a in order to create a 3 × 3 pixels window. After that, the parallel convolution engine, whose C++ HLS description is depicted in Figure 13b, performs the two convolutions centered on the generic pixel P_XY_. The module requires the *#pragma HLS PIPELINE II=1* to assure that the synthetized hardware performs the operations described in the two loops of Figure 13b with a throughput of one clock cycle. The gradient direction is quantized into only four values (i.e., 0, 45, 90, and 135 expressed in sexagesimal degrees) corresponding to four directions: horizontal, bottom–left to upper right, vertical, and bottom–right to upper left. Successively, the output pixel, characterized by two values MAG and DIR, enters the non-maximum suppression module. The latter is composed by a line buffer, as the one depicted in Figure 13a building a 3 × 3 window, followed by a combinatorial module that compares the MAG value of the central pixel P_XY_ (P_XY__MAG) with the MAG values of the two pixels placed on the direction of P_XY_ (P_XY__DIR). If P_XY__MAG is lower than one of them, P_XY__MAG is set to 0, otherwise it keeps its original value. Figure 14 depicts an excerpt of the C++ HLS description related only to the horizontal direction. The obtained value (NMS) is then compared with a low (LT) and a high threshold (HT): if NMS is higher than HT, the pixel is considered to belong to a strong edge and its value is set to 255; if NMS is lower than LT, the pixel is not considered to belong to any edge and its value is set to 0; if any of the above conditions are not verified, the pixel is considered to belong to a weak edge and its value is set to 1. Finally, the weak edge information is converted into a strong edge one if the pixel is spatially adjacent to any pixel classified as strong edge (the C++ HLS description of the last two steps is not reported for the sake of brevity).

Once the output image of the Canny edge detection module is stored back into the external DDR memory, the software routine running on the PS performs the contour selection computational step. At this time, the elaborated image is segmented, as shown in Figure 3c. Then, the DMA_1_ is programmed and activated and the segmented image is sent, through the AXI4-stream protocol, to the second hardware accelerator implementing the module chain Dilate_Erode and Erode_Dilate. To implement such modules, we used the synthesizable intellectual properties (IPs) of the Xilinx *hls_video* library [30]. The modules perform the morphological filtering and send back the output image, depicted in Figure 3d, to the external DDR memory. Finally, the software running of the PS completes the image elaboration with the connected component analysis, to find the geometrical center of the segmented area, and to draw the synthetic ellipse, useful to detect a potential rotation. 

**Figure 15:**
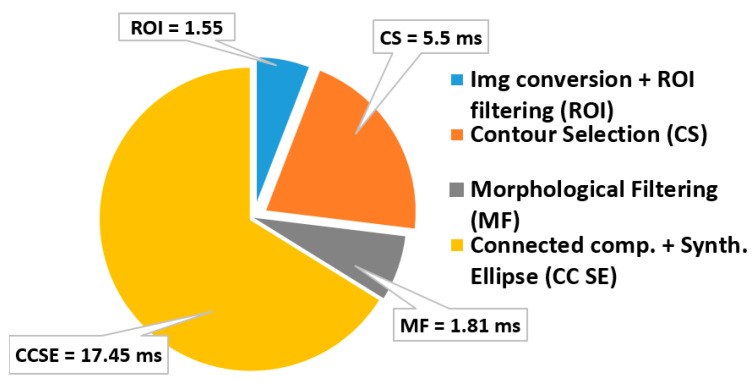
The time breakdown of the geometrical inspection algorithm when running on the embedded system of Figure 11.

**Figure 16:**
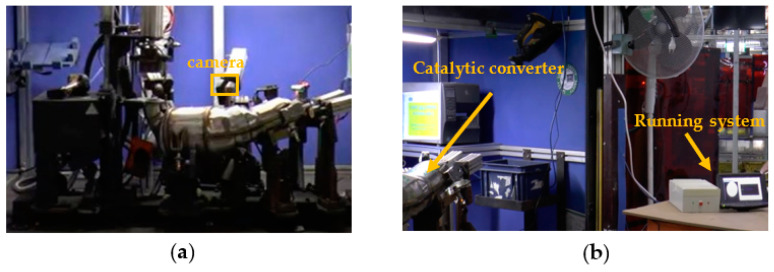
(**a**) The integration of the proposed embedded system into the quality cell and (**b**) a phase of the testing procedure.

Figure 15 shows the timing breakdown when the algorithm runs on the proposed embedded system of Figure 11. It can be seen that the computational time per frame is reduced to only 26 ms, thus resulting in a global computational delay on 50 consecutive frames of only 1.3 s. Compared to the pure software-based solution, the proposed approach shows a speed-up of 23×. Such a high computational speed allows performing the geometrical check of the assembled item without practically adding any extra delay to the existing manufacturing line. As a result, the proposed methodology turns out to be very effective at designing low-cost digital automation systems, to be integrated in the existing productive processes without requiring complex re-engineering, and to boost the well-known Industry 4.0 and smart manufacturing transformation. Figure 16 depicts the integration of the embedded system into the quality cell of the real manufacturing process during the in-line testing phase.

Table 1 reports the hardware resources utilized by the designed accelerators in terms of look-up tables (LUTs), block RAMs (BRAMs), digital signal processing elements (DSPs), and flip-flops (FFs). As it can be inferred, the proposed HW/SW co-designed system has a reduced hardware footprint, so it can be easily embedded in a heterogeneous computing platform with a reduced hardware capability. Table 2 compares area and speed characteristics of the new system with the FPGA-based heterogeneous architecture proposed in [31] to accelerate the bolt inspection task. Despite the different referenced use-cases, compared designs share common CV elaborations, thus allowing a relatively fair analysis. In such a case, even though the system [31] accelerates a lower number of CV operations and it is implemented on a high-end 16 nm FINFet Ultrascale+ device, it achieves a throughput just 39% higher than the proposed architecture. Noteworthy, the latter occupies 67%, 57.7%, and 77.7% less LUTs, FFs, and BRAMs, respectively, thus confirming its suitability for platforms having a reduced footprint.

Finally, Table 3 furnishes an overview of the previous works dealing with computer vision algorithms for inspection tasks. Although the referenced works are related to different application scenarios, algorithms, and implementation platforms, thus preventing a direct comparison with the proposed hardware/software co-design, Table 3 shows that the speed performances achieved by the heterogeneous embedded system here presented are comparable or even better than those reached by more expensive and less integrable computing platforms. The approach presented here demonstrates the feasibility of low-cost and high-performance automated systems for quality inspection tasks that can be easily integrated in area- and time-constrained manufacturing lines. Such a strategy could show further advantages when two or more independent inspection points have to be checked, each one by a specific camera. In these cases, multiple hardware custom processors can be accommodated on the same platform to operate in parallel.

## 5. Conclusions and Future Works

This paper proposes a hardware–software co-design approach to implement an efficient embedded system based on heterogeneous MPSoCs for automatic quality inspection in assembly processes. The high computational speed required by the existing manufacturing line is obtained by purposely-designed FPGA-based hardware accelerators that accelerate the computations of the most timing consuming tasks of the developed computer vision algorithm. When applied to a real assembly process, the proposed methodology resulted in a 23× speed-up compared to a pure software-based solution, thus proving to be a valid solution to design low-cost, high-speed, and compact digital automation systems for the Industry 4.0 transformation. 

The obtained results can pave the way for future research directions. Indeed, with the goal to build a more sophisticated digital twin of the assembled item, the proposed HW/SW design methodology can be exploited to implement more complex image processing routines. As an example, stereoscopic techniques can be useful to automatically detect the distance between the cameras and some points of interest of the assembled item, thus allowing the realization of a more advanced 3D virtual model.

## Figures and Tables

**Table 1:** Hardware resource utilization.

Hardware Component	LUTs	FFs	DSPs	BRAMs (36 Kb)
DMA_0_	1671	2322	0	3
DMA_1_	1487	1948	0	3
RGB2Gray	119	169	3	0
MedianBlur	4795	3383	0	1
Canny	2347	2455	2	3
Dilate_Erode	844	904	0	3
Erode_Dilate	844	904	0	3
AXIS width converters	154	366	0	0
Systems Interrupt	168	159	0	0
PS7 AXI Peripheral	727	1284	0	0
AXI SMC	3495	4316	0	0
TOTAL	16,651	18,210	5	16
% of the available	31.3%	17.1%	2.2%	11.4%

**Table 2:** Comparison with the FPGA design for bolt inspection in [31].

	[31]	This Work
**CV operations**	Threshold, Morphological Filtering, Find centers.	Median Filter, Canny Filter, Contour Selection, Morphological Filtering, Connected Component Analysis.
**Device**	XCZU7EV	XC7Z020
**LUTs**	50,607	16,651
**FFs**	43,012	18,210
**DSPs**	0	5
**BRAMs (36 Kb)**	72	16
**Mpixels/s**	15.7	11.3

**Table 3:** Comparison with prior works.

Work	CV Operations	Time (ms)	Platform	Implementation
[8]	Canny, Morphological Filtering	-	PC	SW
[13]	Sobel edge detection, OTSU thresholding.	-	PC	SW
[14]	Contours Selection, Method of Moments, Template Matching.	~65	-	SW
[15]	Histogram Equalization, Gaussian Filtering, Canny, Circle detection, Inference on a DNN.	33	GPU	SW
[19]	Pixel Thresholding, Median Filter.	250	PC	SW
[21]	Canny, Hough transformation, Background Subtraction.	400	PC	SW
[32]	Contrast enhancement, Gamma transformation, Custom 3 × 3 Convolution.	250	-	SW
[33]	Template Matching.	99	PC	SW
[34]	Median Filter, OTSU thresholding	22.5	PC	SW
[35]	Image Adaptive Thresholding, Find Contours, Template matching, Hough Transform.	-	PC	SW
[36]	OTSU Thresholding, Blob analysis, Morphological Filtering, 3D rendering.	-	-	SW
This work	Median Filter, Canny Filter, Contour Selection, Morphological Filtering, Connected Component Analysis.	26	Xilinx Zynq XC7Z020	HW/SW codesign
